# Automatic Electrodiagnosis of Carpal Tunnel Syndrome Using Machine Learning

**DOI:** 10.3390/bioengineering8110181

**Published:** 2021-11-10

**Authors:** Konstantinos I. Tsamis, Prokopis Kontogiannis, Ioannis Gourgiotis, Stefanos Ntabos, Ioannis Sarmas, George Manis

**Affiliations:** 1 Department of Neurology, University Hospital of Ioannina, 45110 Ioannina, Greece; gourgiotisg@gmail.com (I.G.); ntamposstefanos@gmail.com (S.N.); sarmasioannis@yahoo.gr (I.S.); 2 Department of Physiology, Faculty of Medicine, School of Health Sciences, University of Ioannina, 45110 Ioannina, Greece; 3 Department of Computer Science and Engineering, School of Engineering, University of Ioannina, 45110 Ioannina, Greece; konteap@gmail.com

**Keywords:** carpal tunnel syndrome, CTS, feature extraction, machine learning, median nerve mononeuropathy, nerve conduction studies

## Abstract

Recent literature has revealed a long discussion about the importance and necessity of nerve conduction studies in carpal tunnel syndrome management. The purpose of this study was to investigate the possibility of automatic detection, based on electrodiagnostic features, for the median nerve mononeuropathy and decision making about carpal tunnel syndrome. The study included 38 volunteers, examined prospectively. The purpose was to investigate the possibility of automatically detecting the median nerve mononeuropathy based on common electrodiagnostic criteria, used in everyday clinical practice, as well as new features selected based on physiology and mathematics. Machine learning techniques were used to combine the examined characteristics for a stable and accurate diagnosis. Automatic electrodiagnosis reached an accuracy of 95% compared to the standard neurophysiological diagnosis of the physicians with nerve conduction studies and 89% compared to the clinical diagnosis. The results show that the automatic detection of carpal tunnel syndrome is possible and can be employed in decision making, excluding human error. It is also shown that the novel features investigated can be used for the detection of the syndrome, complementary to the commonly used ones, increasing the accuracy of the method.

## 1. Introduction

**Figure 1:**
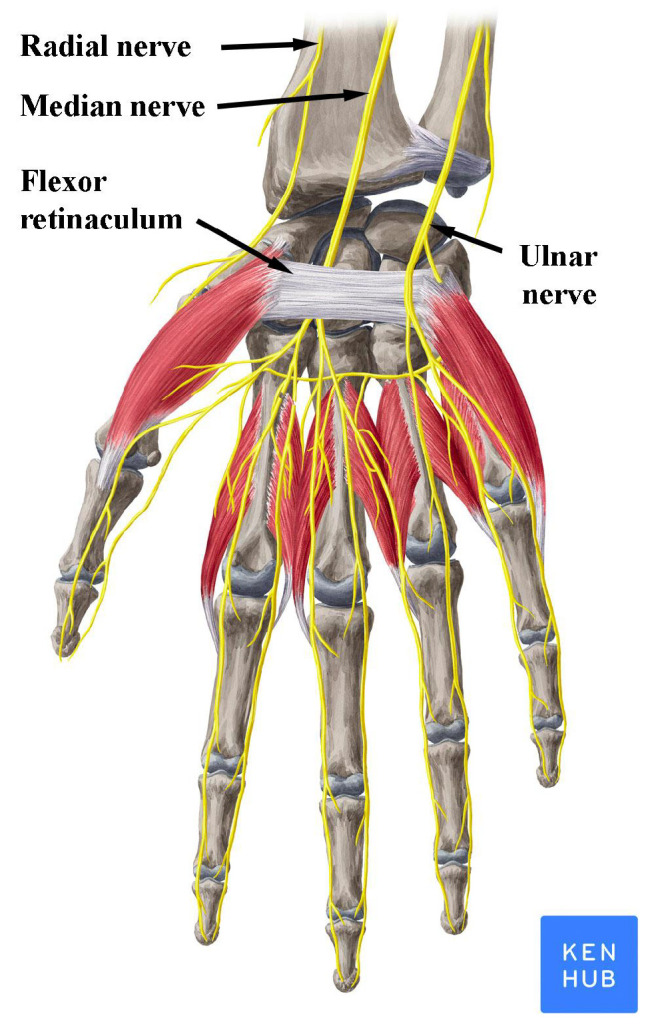
Nerves of the wrist and hand. Note the position of the median nerve under the flexor retinaculum. Reproduced with permission from www.kenhub.com, accessed on 1 August 2021.

*Carpal Tunnel Syndrome* (*CTS*) is caused by compression of the median nerve as it travels through the carpal tunnel, formed by flexor retinaculum, while the other nerves of the hand (ulnar and radial) are not affected by the syndrome (Figure 1). It is the most common peripheral compression mononeuropathy and a significant cause of work disability, with almost 3% of the general population affected [1,2]. Compressing forces on the median nerve result in microvascular damage to the nerve and its myelin sheaths. Prolonged or repetitive compression results in inflammation, fibrosis and demyelination, which leads to disruptions in the speed of axonal signaling [3]. With persistent compression, the combination of these factors may lead to axonal degeneration [4]. The symptoms that define the syndrome are numbness and tingling in the median nerve distribution along with pain at the palmar side of the hand and the wrist. Symptoms typically start insidiously, during the night and they gradually exacerbate. These initial sensory symptoms are followed by weakness in grip and atrophy of thenar muscles if the condition remains untreated [5]. Moreover, the pain is frequently reported to spread proximally to the wrist up the arm and sensory symptoms are often described outside the typical median nerve distribution [6,7].

Clinical diagnosis of CTS is made with an accurate patient history describing a combination of the typical symptoms, along with physical examination of the hand and clinical provocative tests (Tinel’s sign, Phalen’s test and compression test) [8]. Final diagnosis and decisions about the further treatment of CTS is accomplished by the detection of median nerve mononeuropathy through the use of electrophysiological and imaging studies [9,10]. Imaging modalities, including magnetic resonance imaging and ultrasonography of the wrist, had low sensitivity and conflicting results, until recently, and they were not recommended for routine use in diagnosis [8]. However, improvements in ultrasound equipment and the training of physicians increased its sensitivity and specificity in the detection of median nerve mononeuropathy, making it a useful tool for diagnosis, as well as for the detection of anatomical abnormalities, contributing to median mononeuropathy [10,11,12,13]. Magnetic resonance imaging is a useful tool for the assessment of various traumatic and pathological conditions in the wrist and hand, including nerve mononeuropathy [14,15], but its routine use is not supported for the diagnosis of CTS [10].

On the other hand, electrophysiological *Nerve Conduction Studies* (*NCSs*) have long been considered the most useful tool for the confirmation of the median nerve mononeuropathy and the decision of therapeutic strategy in CTS [5]. Analysis of the median nerve motor and sensory conduction across the wrist compared to ulnar and radial nerve conduction is used to evaluate the functional result of median nerve compression [16]. Different parameters with comparable high sensitivities are included in NCSs depending mainly on the neurophysiologist’s experience [17]. The combination of these commonly used parameters for the neurophysiological testing, has led to the development of a grading scale widely accepted for the diagnosis and management decisions [18].

Nevertheless, many controversies have arisen regarding the necessity of NCSs as confirmatory tests or tools contributing to patient management [19,20,21,22]. These controversies emanate mainly by the rise of the time and the cost needed for the diagnosis of CTS with the use of NCSs [23,24], the discomfort caused to the patients by nerve stimulation, and the high rate of false positive and false negative results of NCSs along with the ease and the accuracy of clinical diagnosis in many cases with symptoms and signs typical of CTS [20]. Thus, the present study sought out to test the sensitivity of the different NCSs’ parameters for the diagnosis of CTS with a final goal to facilitate and accelerate the electrodiagnosis and make it more accurate.

Recently, machine learning based methods have found widespread use in healthcare, revolutionizing the area of medical diagnosis [25,26,27]. To this end, previous attempts for the automatic diagnosis of neuropathies and CTS through machine learning NCS signal processing have shown promising results [28,29]. However, these studies are focused exclusively in the analysis of motor nerve conduction parameters, having — in the case of CTS — the inherent drawback of the wide variation of compound muscle action potentials (CMAP) related to muscle contraction and hand position [30]. Furthermore, there is a number of hand-held devices developed for the diagnosis of peripheral neuropathies, but they employ analysis solely of specific signal characteristics carried out with restricted numbers of classifiers [31,32], resulting in very limited use in the diagnosis of CTS, indispensably together with thorough history and clinical examination [33,34]. This study aims to highlight the accuracy that can be reached in the automatic diagnosis of CTS by the analysis of diverse NCS signal characteristics with multiple classifiers.

## 2. Materials and Methods

The main target of this study is to evaluate the sensitivity of different NCSs’ parameters for the diagnosis of median nerve mononeuropathy and the ability of different classifiers to automatically discriminate normal and pathological values. Machine learning techniques were employed to examine signal characteristics and compute the accuracy of automatic classification.

### 2.1. Data

This study included 38 volunteers that were examined prospectively, over a period of two months, at the Laboratory of Clinical Neurophysiology, Neurological Department of the University Hospital of Ioannina. Among them, 28 participants were patients with symptoms and signs suggestive of CTS, who were referred to the laboratory by hospital physicians not affiliated to the study (neurologists, orthopedics and rheumatologists). Patients diagnosed with other neurological disorders apart from CTS (other neuropathies, radiculopathies, central nervous system lesions), as well as patients with other systemic diseases, were excluded from the study. Ten age-matched healthy individuals were also included in the study (Table 1).

All participants signed an informed consent form and underwent an initial clinical evaluation from two neurologists of the study for both hands, before neurophysiological testing. From the total number of hands examined (76), 65 were included in the study (46 with CTS and 19 control) and 11 hands were excluded because there was either a history of prior surgical intervention for CTS or a mismatch between clinical and neurophysiological diagnosis [13,35]. Clinical diagnosis of CTS for each hand was based on the following criteria: (a) hypesthesia or paresthesia in the distribution of the median nerve; (b) night paresthesia; (c) wrist and palm pain; (d) weakness or atrophy of the thenar musculature; (e) Tinel’s or Phalen’s signs. Each hand was clinically categorised into three groups. The first group, assigned as control, included 19 hands without any symptomatology. The second group, assigned as mild to moderate, included 27 hands with solely sensory symptomatology and pain. The third group, assigned as severe, included 19 hands that had developed weakness and/or thenar atrophy, apart from the sensory symptoms. Taking into account the inherent subjectivity of sensory symptomatology, we used the emergence of motor symptoms for the clinical categorization, modified from a previously described clinical scale [36].

A detailed neurophysiological examination of both hands was carried out on all participants using the same equipment (Dantec Keypoint, two channel EMG), as previously described [37]. The temperature of the upper limbs to be examined was acclimatized at 30–31 °C. Bilateral median motor nerve distal latency, median motor nerve conduction velocity from the elbow to the wrist, median sensory nerve conduction velocity from the wrist to the second and forth digits and motor and sensory action potential amplitude were measured. Bilateral radial nerve sensory and ulnar nerve motor and sensory conduction studies were also performed, in order to exclude other possible neuropathies and compare parameters to the median nerve. Based on the NCSs’ findings, the hands were categorized into four main groups, using a modification of the neurophysiological grading introduced by Bland JDP [18]. The first group included 19 hands without findings in NCSs suggestive of median nerve mononeuropathy (MM). This is the control group *A* (no MM). Group *B* (mild MM) included ten hands with sensory conduction velocity from index finger to wrist smaller than 40 m/s and motor terminal latency from wrist to *Abductor Pollicis Brevis* (APB) smaller than 4.5 m/s. Group *C* (moderate MM) included 17 hands with motor terminal latency for APB between 4.5 m/s and 6.5 m/s and preserved index finger *SNAP (Sensory Nerve Action Potential)*. Group *D* (severe MM) included 19 hands with motor terminal latency for APB>4.5 m/s and absent SNAP or motor terminal latency larger than 6.5 m/s. With the symbol BCD we refer to the union of sets *B*, *C* and *D*. The participants of the study were carefully selected and each hand was finally included in the dataset only in the case of a perfect match between clinical diagnosis and NCSs, enabling a robust grouping of the hands and facilitating further accurate signal analysis and classification.

### 2.2. Methodology

Next, machine learning techniques were used to analyze those data and investigate the sensitivity of the signal characteristics and the ability to automatically discriminate controls and patients. Experiments were performed with two sets of features. The first one consisted of those features which are considered common and are widely used today by medical doctors and medical devices for the diagnosis of median nerve mononeuropathy. The purpose was to test whether it is possible to achieve a high level of accuracy in automatic diagnosis using machine learning classifiers and the widely used features. In order to decouple the classification capability from the selected classifiers, experiments were performed with five different classifiers, selected based on their popularity and the expected ability to achieve increased accuracy.

The second set of features consisted of novel features. The purpose this time was to identify new features which could carry useful diagnostic information and do not belong to the set of the common features; features which, if used in addition to the common ones, could possibly increase the classification capability of the machine learning classifiers. Features with physical or mathematical meanings were selected.

Finally, feature selection techniques were employed in order (a) to evaluate the level of significance of each feature in the automatic diagnostic procedure and (b) to help the classifiers present a better performance, a common practice in machine learning.

### 2.3. Signal Features

Features were separated into two subsections; one for the *common* features and one for the *novel*.

#### 2.3.1. Common Features

**Figure 2:**
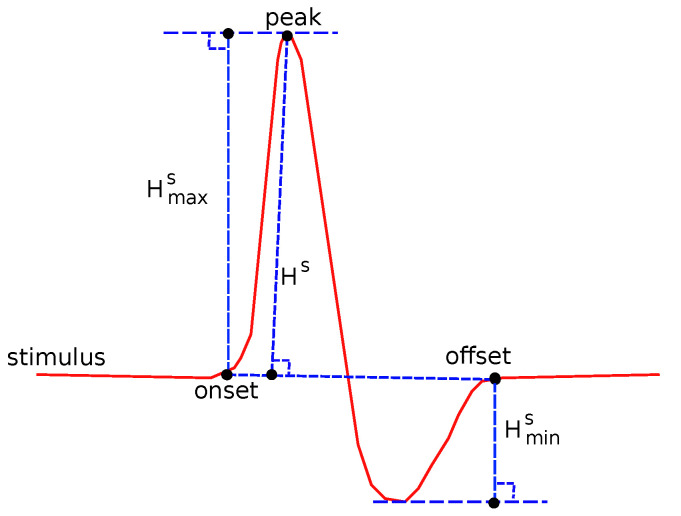
Several features related to definitions about amplitude.

**Figure 3:**
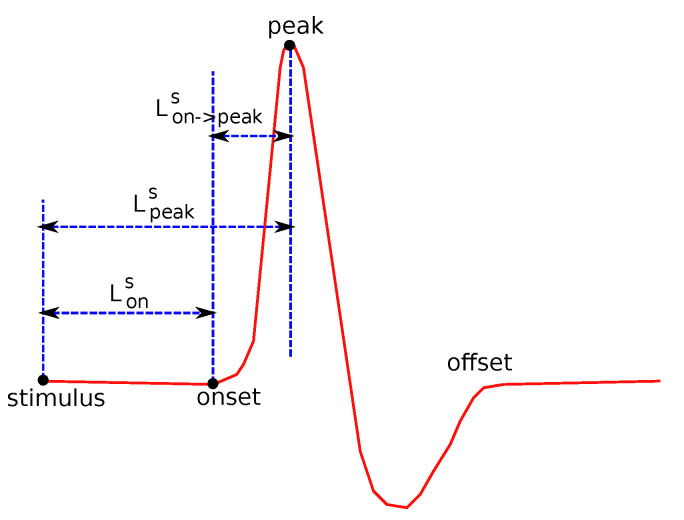
Several features related to definitions about duration.

**Figure 4:**
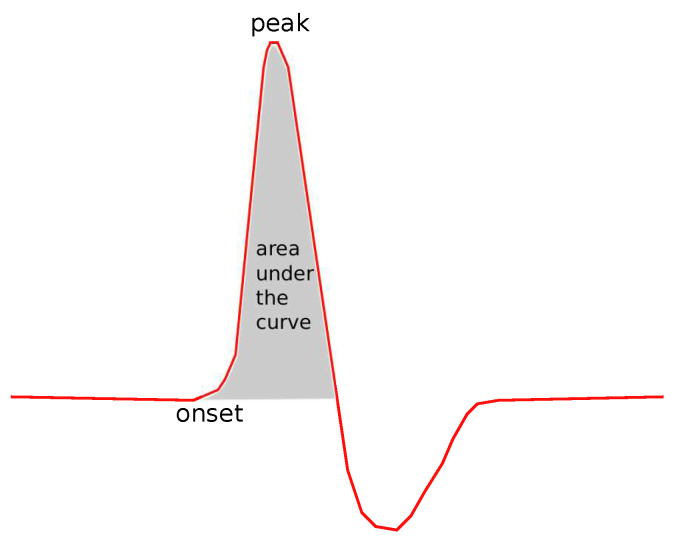
The area under the curve.

The following common features were tested: Sensory Amplitude or Height ($H^{s}$, Figure 2), Sensory Maximum Value Amplitude ($H_{\max}^{s}$, Figure 2), Maximum minus Minimum Amplitude ($H_{\max-\min}^{s}$, Figure 2), Sensory Onset Latency ($L_{\mathrm{on}}^{s}$, Figure 3), Sensory Peak Latency ($L_{\mathrm{peak}}^{s}$, Figure 3), Sensory Onset to Peak Latency ($L_{\mathrm{on}\to \mathrm{peak}}^{s}$, Figure 3), Sensory Conduction Velocity ($CV^{s}$), Sensory Area ($A^{s}$, Figure 4), Motor Distal and/or Proximal Amplitude ($H^{mD}$, $H^{mP}$), Motor Distal and/or Proximal Takeoff Latency ($H_{\mathrm{takeoff}}^{mD}$, $H_{\mathrm{takeoff}}^{mP}$), and Motor Conduction Velocity ($CV^{m}$).

#### 2.3.2. Novel Features

Next, some features are presented, tested for the first time in this study for the diagnosis of CTS. The main goals and motivation to test these features were:

- To investigate characteristics which have some inherent diagnostic information, not exploited by common features;
- To explore features, which may reveal new diagnostic information, not easily predicted or explained by current knowledge. Such features should be further investigated with other studies and examined by a larger number of researchers, before reaching a conclusion about possible physical meaning;
- To suggest features that do not heavily depend on the detection of critical points of the signal. This detection is now performed manually by the medical doctor, who indicates them with the aid of a pointing device. In this way, the extraction process is independent from the manual detection and excludes the human error.

The examined features are described below.

 *(a)* *Expanded Absolute Sensory Area ($|A_{e}^{s}|$):*

**Figure 5:**
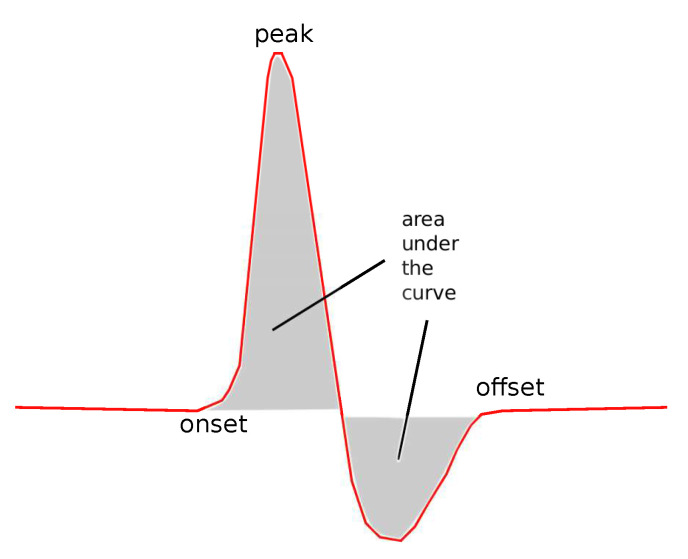
The expanded absolute area.

A descriptive name for this feature is *Expanded Absolute Sensory Area*, a graphical example of which is shown in Figure 5. It consists of both the positive and the negative part of the curve. The negative part is treated as positive, considering the absolute values of the curve. There is a physical meaning behind this feature, since this small negative curve following the positive part of the curve corresponds to the repolarization of the membranes of the cell [38,39].

 *(b)* *Duration ($D^{s}$) and Duration at Half Maximum ($D_{h/2}^{s}$):*

**Figure 6:**
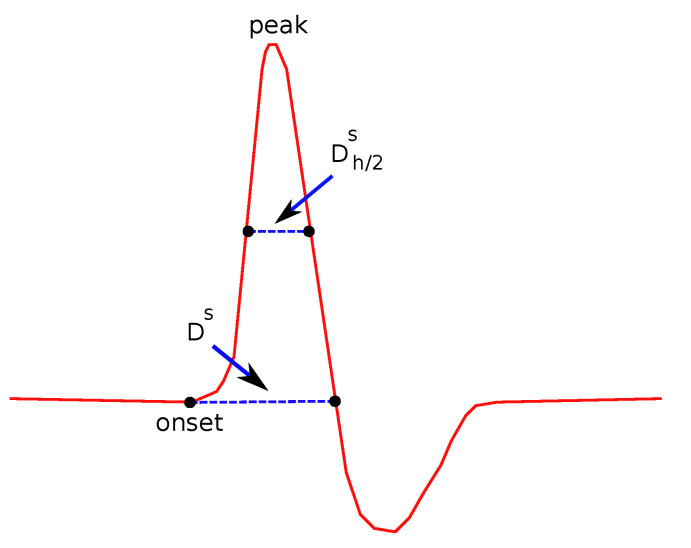
Features related to duration.

$D^{s}$ is defined as the duration (the width in x-axis) of the signal between the onset and offset point of the curve. $D^{s}$ is shown in Figure 6.

$D_{h/2}^{s}$ is defined as the duration of the signal between the points where the y-values are taking the half of the maximum value, as also shown in Figure 6. Compared to $D^{s}$, $D_{h/2}^{s}$ provides additional and complementary information, since it also takes into account the height of the wave as well as its shape, since an acute wave will give a smaller value of $D_{h/2}^{s}$ than a less acute one.

 *(c)* *Areas and their ratios:*

**Figure 7:**
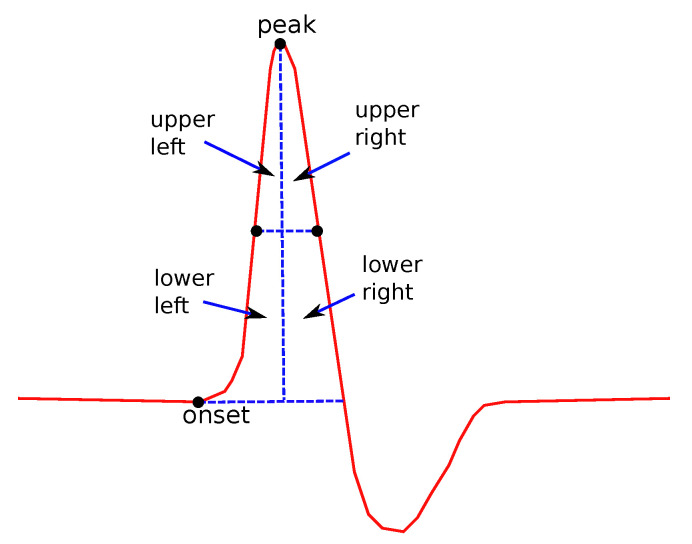
Several features related to definitions about subareas.

In Figure 7, the definition of the four main subareas is presented. The areas are defined by the line between the points where the y-values take the half of the maximum value (used for the definition of $D_{h/2}^{s}$) and perpendicular to the x-axis line, crossing the peak point. The following four subareas are defined: Upper Left Area, Lower Left Area, Upper Right Area an Lower Right Area. From these partitions, the following areas are defined: the Left Area as the sum of Upper and Lower Left areas and the Right Area as the sum of Upper and Lower Right areas, Upper Area as the sum of Upper Left and Upper Right areas and Lower Area as the sum of the Lower Left and Lower Right Areas. All possible ratios between any two of the above areas were also considered as features. In addition, the ratio between each of the above areas over the total area under the curve was also examined.

Areas are of special interest. They not only describe single features as the duration or the height, but they also consider relations between figures, since their values depend on the duration, height and the shape of the curve. Even though sub areas cannot be directly connected to physiology, it cannot be ignored that valuable information can be hidden there. This information is very difficult to explain, since, as noted above, it depends on more than one factor. The rations between areas, especially, could hide information that is even more complicated and difficult connect to physiological reasons.

 *(d)* *Slopes:*

**Figure 8:**
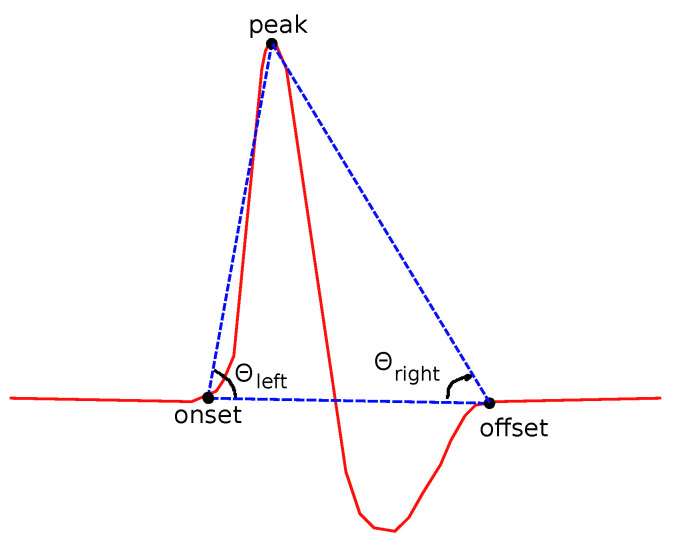
Features related to slopes based on onset, offset and peak points.

Two kinds of slopes have been examined for both left and right sides of the curve. $\theta_{\mathrm{left}}$ is the angle of the line that connects the onset and the peak points (Figure 8):

(1) $$\tan\theta_{\mathrm{left}}=\frac{f_{\mathrm{peak}}-f_{\mathrm{onset}}}{x_{\mathrm{peak}}-x_{\mathrm{onset}}}.$$

A similar definition comes from the right side of each curve:

(2) $$\tan\theta_{\mathrm{right}}=\frac{f_{\mathrm{offset}}-f_{\mathrm{peak}}}{x_{\mathrm{offset}}-x_{\mathrm{peak}}}.$$

**Figure 9:**
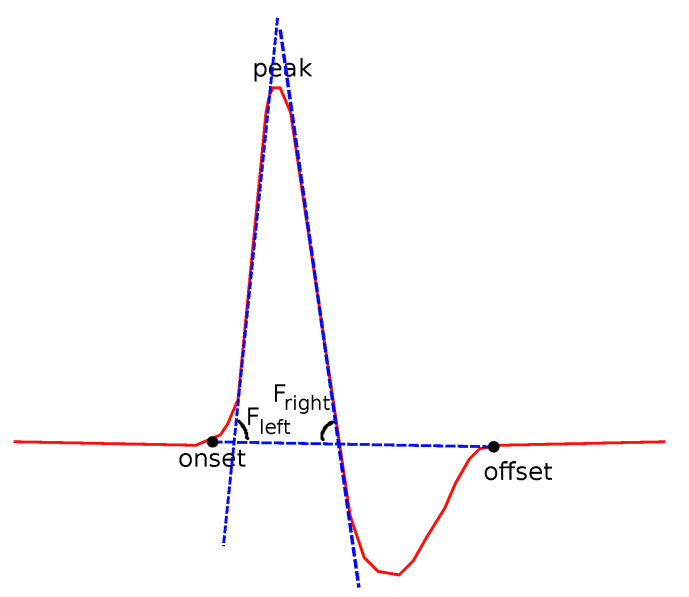
Features related to slopes based on regression.

The fitting slope is also an interesting feature. The *Fitting slope* is the line that best fits all the data points of the left ($F_{\mathrm{left}}$) and then of the right ($F_{\mathrm{left}}$) sides of the curve, using regression analysis (Figure 9). The fitting slope is of special interest, since its computation is almost independent from the human error. A reasonable lack of accuracy in the detection of the onset and offset points does not affect the computed value of $F_{\mathrm{left}}$ or $F_{\mathrm{right}}$.

### 2.4. Processing

The level of noise was much higher in sensory than in motor signals. A moving window average technique was used to smooth the signal and a detrending one to remove the effect of isoelectric line drift.

Critical points were detected on the smoothed signal and were then projected back to the original one. The peak point and the lowest one were first and easiest to locate using maximum and minimum values in a time frame after the initial stimulus. Then, starting from the peak point, and moving backward, the onset point was detected, exploiting the sharp change of the slope in this part of the curve. The slope threshold was set experimentally, common for all signals. In a similar way, the computation of the offset started from the lowest point and moved forward until the change in the slope indicated the offset point. The detection of the offset point and the peak and lowest points was more accurate than the estimation of the offset point, something that was concluded after visual inspection.

The critical points were used for the computation of the features discussed earlier, in Section 2.3. Features were fed as input to five classifiers. State-of-the-art machine learning algorithms were selected: Decision Trees (DT), Support Vector Machines (SVM), k-Nearest Neighbors (kNN), Naive–Bayes (NB) and Logistic Regression (LR). Thirty four features were defined (common and novel) and each feature was computed for eight different signals of every hand. These signals include the sensory conduction of the median nerve on the second and fourth digits, the sensory conduction of the ulnar nerve on the fourth and fifth digits, and the motor conduction of median and ulnar nerves on proximal and distal stimulation. So, the total number of features extracted from each hand is 34×8=272, which is a very large number of features for a classifier. If 30 features were added to this number, which were computed as differences of some of those 272 features, a total number of 302 features is reached.

Thus, in order to reduce the number of features and assist the classifiers to optimize their accuracy, feature selection and parameter tuning were applied. Given the large number of features and the relatively small number of recordings, a custom feature selection and parameter tuning technique was selected.

All classifiers were trained and tested separately with one feature as input each time and for a reasonable range of parameters. For example, for the 3-class problem and for SVM with an RBF kernel, the values 0.1≤c≤3 were checked with step 0.1, a range that seemed adequate enough, according to the variation of accuracy values computed. The value c=0.9 seemed to be the best one, based on the number of features presenting high accuracy with this value. For NN, and the same problem, the values 1≤nn≤11 were checked and the best value appeared to be nn=5.

Using the selected parameters, each classifier was trained again. A custom *sequential feature selection* technique was applied, using NN and SVM. The custom feature selection technique was based on the *sequential backward feature selection (SBS)* and the *sequential backward feature selection (SFS)* algorithms [40]. In each step of SBS, the feature causing the maximum performance loss gets removed, while in each step of SFS the feature adding the maximum to the performance is added. The custom version combines those two algorithms and consists of two phases. In the first phase, an initial set of features consisted of all extracted features was considered. After each time excluding one of the features from this set, the performance of both classifiers was tested. If none of the two classifiers had improved its performance, the specific feature was excluded from the set. In the second phase, the initial set was defined as the set with those features which were not eliminated during the first phase. Starting by adding, one by one, each excluded feature, and following the same order as the previous phase, the performance of the two classifiers was tested again. Those features that increased the performance of at least one classifier were considered significant.

For training and testing leave-one-out cross validation was selected, a technique appropriate when the amount of available data is not large. *K*-fold cross validation is called the technique of partitioning the dataset into *k* subsets of equal size. One of these subsets is used as test data and the other k−1 subsets are used as training data. The validation process is being repeated *k* times, since each subset must play the role of the test data exactly once. In the special case where k=N (*N*: number of instances), the *k*-fold cross validation is induced to leave-one-out cross validation.

## 3. Results

Experiments were performed with five classifiers (*LR, SVM, kNN, DT, NB*) in order to decouple the classification capability of each classifier from the results. The dataset was segregated into several subsets and different problems were studied based on this segregation. Their names were given based on the number of the classes that were attempted to be discriminated: *4-class*, *3-class* and *2-class*. In the *4-class* problem, the categorization between the groups *A* (no MM), *B* (mild MM), *C* (moderate MM) and *D* (severe MM) was studied. Please also see Section 2 for the definition of those subsets. In the *3-class* problem, the groups *B* and *C* were merged and the categorization between groups *A*, BC (mild and moderate) and *D* (severe) was studied. Finally, in the *2-class* problem, all groups with symptoms (*B*,*C*,*D*) were merged into one BCD and controls (*A*) and patients (BCD) were compared.

The configuration for each classifier was different for every problem and the parameters were selected after experimentation. For example, for the 3-class and 4-class problems, the RBF kernel was proved best for the SVM classifier with C=0.9. For the 2-class problem, the linear SVM presented the most accurate classification. When the NN classifier was used, the best results were achieved for nn=5.

For all problems, the common features and the novel features were studied separately and how all features work together was investigated, in comparison with the standard neurophysiological diagnosis of the physicians with NCSs. There is, however, an inevitable bias on common features, given that the electrodiagnosis has been based on them.

The same experiments were performed on the clinical grading. The purpose of these experiments was to evaluate all features in correlation with clinical symptoms and not just to the electrodiagnosis. Clinical evaluation is considered the gold standard for the diagnosis of CTS and does not always correlate absolutely with NCSs’ findings. Thus, the comparison of common and novel features upon the field of clinical grading is necessary and very interesting.

Results are presented in Table 2, Table 3 and Table 4. On the third column, the classification accuracy of the common features is shown. Bold letters indicate the best performing classifier.

As *accuracy*, we consider the fraction of the successfully classified subjects over the total number of subjects:

$$accuracy=\frac{successfully\_categorized\_subjects}{all\_samples}=\frac{true\_possitive+true\_negative}{all\_samples}.$$

The accuracy of the novel features can be found in the fourth column and in the classification of all features in the last column. Each line of a table corresponds to a different classifier. As described in Section 2, clinical categorization classified the study population into three groups, so it was not included in the 4-class problem experiments. For the 3-class and the 2-class problems, comparisons with the clinical symptoms were also included and are shown in the lower part of the tables.

For the 4-class problem presented in Table 2, one can notice that the novel features achieved a much lower accuracy. This is expected, not only for the 4-class problem, but also for the 2 and 3-class problems. It should be emphasized here that subjects have been classified by the medical doctor using the common criteria. So, there is a bias in the comparison between common and novel features against the novel ones, since the system is trained with expertise extracted with the common criteria. This is why in Table 2, Table 3 and Table 4 the common criteria present better categorization. However, when both common and novel criteria are used as inputs to the classifier, the total accuracy (last column in these tables) is almost always improved, indicating the benefits of using both common and novel criteria in the automatic electrodiagnosis.

In the 3-class problem (Table 3), the novel features also ameliorated the performance of the models if put together with the common features. Apart from that, they performed better than the common features in the field of clinical grading for most classifiers. This is also very important, if one considers that the medical doctors’ diagnosis has been based mainly on the common features.

With the experiments comparing patients to control group (Table 4), which correspond to the 2-class problem, the addition of the novel features could also ameliorate the performance of most classifiers. It also performed better on the field of clinical grading than the common features for most of the classifiers.

From all three tables, one can notice that SVM presents the highest accuracy among all examined classifiers, with a small insignificant exception. The inclusion of novel characteristics improved SVM’s performance in almost all cases. In the field of clinical grading, the novel features performed better than the common features, and improved the performance of SVM both in the 3-class and the 2-class problems.

In Table 5, the accuracy of two of the classifiers is presented, when a single feature is used as an input. This table shows the effectiveness of each feature, which can be compared with the entire feature set for each feature category (Table 2, Table 3 and Table 4). Since the number of the features used was very large, the table includes only the features selected by the feature selection process and some of the best, but not selected, features from each group.

Finally, confusion matrices (Table 6) from the two class problem and the best performing classifier are presented. The best performing classifier was the DT for the common features and SVM when the novel or all features were used. Matrices show how many hands which were predicted positive/negative were actually positive/negative. For example, in the third matrix of Table 6, for the 44 hands predicted positive, all 44 were actually positive and from the 21 hands predicted to be negative, 19 were actually negative, but two were positive. This corresponds to a rate of 1 and 0.995, respectively. Looking at the matrix from the other side, from the 46 hands which were actual positive, two of them were predicted to be negative and 44 positive with a success rate of 0.957. All actual negative hands were predicted correctly (rate:1).

## 4. Discussion

Recent literature has revealed a long discussion about the importance and necessity of NCSs in CTS management. Laboratory findings supporting clinical diagnosis are necessary for decision making about further management in CTS, especially when invasive treatment is needed [41]. To date, the most reliable diagnostic tool for CTS is electrodiagnosis, but its usability is being questioned due to the rise in the time and the cost needed for the diagnosis, with the employment of NCSs, as well as the emergence of ultrasonography as an alternative [23,24]. In this study, the parameters of the NCSs that are currently widely used for the diagnosis of median nerve mononeuropathy were evaluated. New features of the waveforms that could ameliorate the accuracy of NCSs were tested. These features describe the right side of the signal curves that is related to the repolarization of axons. The repolarization processes can be affected in nerve injury [42] and current diagnostic use of NCS for CTS does not include these parameters.

Machine learning algorithms with five classifiers (LR, SVM, kNN, DT, NB) were employed in the NCS signal analysis and the results were compared to the neurophysiological and clinical diagnosis. Automated discrimination between patients and controls, with the most accurate classifier (SVM), reached an accuracy of 0.9513 compared to NCS and 0.8906 compared to clinical diagnosis. Further analysis with SVM and classification into three groups (control group, patients with mild and moderate CTS, patients with severe CTS) reached an accuracy of 0.9415 compared to NCS and 0.7144 compared to clinical diagnosis. Finally, SVM performed equally well in the classification of the study population into four groups (control group, patients with mild CTS, patients with moderate CTS and patients with severe CTS), with an accuracy of 0.9117 compared to NCS. Comparing the maximum accuracy of the present study with other studies in the literature [28,32,43], it is evident that the methodology described here has superior results, keeping in mind that the comparison concerns studies with differences in the dataset and the experimental configuration.

Currently, NCS is considered the gold standard for the confirmation of median nerve mononeuropathy in the CTS. The aim of the present study is to find a methodology to enable automatic diagnosis through the analysis of conventional electrodiagnosis signals with machine learning techniques. This would facilitate and accelerate the electrodiagnosis, excluding the human error. The results show that the inclusion of the novel features described here can increase the accuracy of the automatic electrodiagnosis, when they are used together with the common features. So, the inclusion of the novel features does not aim to replace the common features but to enhance their performance.

## 5. Conclusions

The findings of this study show that an accurate automatic electrodiagnosis for median nerve mononeuropathy is possible. Apart from testing the accuracy of the common NCSs’ features, several geometric features with indicated physiological meaning for median nerve compression were extracted and presented. These features are employed for the first time in the workup of CTS. The classification model proposed is unbiased and indicates that these features can play an important role in supporting the diagnosis of the syndrome and grading its severity. However, the evaluation of the results through a larger database of CTS patients would be useful, in order to establish an accurate grading scale and produce more robust generalizations. Such a grading scale could serve as a basis for an automatic electrodiagnosis, excluding the human error, implemented in costume electrophysiological testing or hand-held devices.

## Figures and Tables

**Table 1:** Characteristics of the study population.

Characteristics ${}^{1}$	Patients (*n* = 28)	Controls (*n* = 10)
Women	25 (89%)	7 (70%)
Age (years)	47.93±12.92	41.3±9.14
Weight (kgr)	72.55±13.97	66.50±10.23
Height (cm)	163.35±7.68	166±8.94
BMI ${}^{2}$ (kg/cm${}^{2}$)	27.23±5.15	24.13±3.16

${}^{1}$ There is no statistically significant difference between the groups. ${}^{2}$ Body Mass Index.

**Table 2:** Four-class Problem Classification Accuracy.

		Common	Novel	All
NCS	LR	0.7123	0.5455	0.7792
	SVM	**0.8243**	**0.7388**	**0.9117**
	kNN	0.6623	0.5714	0.7403
	DT	0.7068	0.6364	0.8182
	NB	0.8092	0.7143	0.8701

**Table 3:** Three-class Problem Classification Accuracy.

		Common	Novel	All
NCS	LR	0.8172	0.6364	0.8312
	SVM	**0.9404**	**0.8622**	**0.9415**
	kNN	0.7208	0.6654	0.7922
	DT	0.8052	0.8302	0.8117
	NB	0.9013	0.7403	0.8961
Clinical	LR	0.5195	0.5909	0.5519
	SVM	**0.6867**	0.7187	**0.7144**
	kNN	0.5395	0.5169	0.5610
	DT	0.5539	**0.7273**	0.5649
	NB	0.6549	0.5642	0.6594

**Table 4:** Two-class Problem Classification Accuracy.

		Common	Novel	All
NCS	LR	0.8052	0.8182	0.8377
	SVM	0.9513	**0.9077**	**0.9692**
	kNN	0.8471	0.8442	0.8805
	DT	**0.9538**	0.8701	0.9481
	NB	0.9481	0.8142	0.9091
Clinical	LR	0.7957	0.8247	0.7403
	SVM	**0.8691**	**0.8748**	**0.8906**
	kNN	0.7403	0.8312	0.8701
	DT	0.7792	0.7438	0.8571
	NB	0.8532	0.7926	0.8172

**Table 5:** Accuracy achieved by each feature separately.

	kNN			SVM		
from feature selection:	2-class	3-class	4-class	2-class	3-class	4-class
Median Sensory Onset Latency Digit 4	0.89	0.84	0.81	0.88	0.84	0.78
Median Sensory Conduction Velocity Digit 4	0.87	0.79	0.74	0.84	0.81	0.75
Median Sensory Max Amplitude Digit 4	0.82	0.58	0.50	0.81	0.59	0.58
Median Motor Take-off Latency	0.87	0.75	0.66	0.85	0.78	0.73
Median Motor Right Area	0.66	0.53	0.52	0.75	0.51	0.50
Median Sensory Right Tangent Theta Digit 4	0.76	0.63	0.62	0.76	0.69	0.64
Med/Uln Difference Sensory FDHM Digit 4	0.64	0.34	0.26	0.66	0.42	0.25
common features:	2-class	3-class	4-class	2-class	3-class	4-class
Median Motor Amplitude	0.57	0.32	0.40	0.66	0.40	0.39
Median Sensory Area Digit 4	0.74	0.48	0.39	0.72	0.55	0.48
novel features:	2-class	3-class	4-class	2-class	3-class	4-class
Median Motor Left Area	0.67	0.42	0.35	0.68	0.38	0.37
Median Motor Left Slope	0.75	0.59	0.53	0.71	0.62	0.60
Median Motor Right Area	0.75	0.43	0.33	0.70	0.49	0.30

**Table 6:** Confusion matrices for the 2-class problem.

DT/Common	Predicted Positive	Predicted Negative	
**Actual Positive**	44	2	0.956
**Actual Negative**	1	18	0.947
	0.978	0.900	0.9538
**SVM/Novel**	**Predicted Positive**	**Predicted Negative**	
**Actual Positive**	41	5	0.891
**Actual Negative**	1	18	0.947
	0.976	0.783	0.9077
**SVM/All**	**Predicted Positive**	**Predicted Negative**	
**Actual Positive**	44	2	0.957
**Actual Negative**	0	19	1
	1	0.995	0.9692

## Data Availability

All NCSs data of the study are freely available upon reasonable request.

## Abbreviations

The following abbreviations are used in this manuscript:

CTS	Carpal Tunnel Syndrome
NCS	Nerve Conduction Studies
MM	Median Nerve Mononeuropathy
kNN	k-Nearest Neighbors
DT	Decision Trees
SVM	Support Vector Machines
NB	Naive–Bayes
LR	Logistic Regression
FDHM	Full Duration Half Maximum
